# Clinicoradiological Profile of Patients Having Drug-Sensitive Pulmonary Tuberculosis With and Without Diabetes Mellitus in a Tertiary Care Hospital in Ahmedabad, Gujarat, India

**DOI:** 10.7759/cureus.58810

**Published:** 2024-04-23

**Authors:** Anuj Barot, Asmi Vora, Om Dobariya, Vraj Parikh, Loolu Rahumath S, Nalin Shah, Ghanshyam Borisagar

**Affiliations:** 1 Internal Medicine, Byramjee Jeejeebhoy Medical College, Ahmedabad, IND; 2 Respiratory Medicine, Byramjee Jeejeebhoy Medical College, Ahmedabad, IND

**Keywords:** drug-sensitive tuberculosis, chest x-ray, tuberculosis, diabetes mellitus, pulmonary tuberculosis

## Abstract

Background

A significant effect of diabetes mellitus (DM) on the clinical and radiological features of tuberculosis (TB) has been reported. However, conflicting results have also been reported. Hence, a conclusion is yet to be drawn. This study aimed to analyze and compare the clinical and radiological features of drug-sensitive pulmonary TB with DM and without DM.

Methodology

A comparative, observational study was conducted between August and October 2023. Patients with drug-resistant TB, extrapulmonary TB, those on immunosuppressive drugs, and human immunodeficiency virus-positive individuals were excluded from this study. Pulmonary TB patients with DM were classified as the case group and those without DM were classified as the control group. Demographic details, clinical symptoms, detailed past and family histories of comorbid conditions, laboratory investigations, sputum acid-fast bacilli results, and chest X-ray findings were noted. The diagnosis of TB and grading of sputum smear results were done by following the National Tuberculosis Elimination Program guidelines.

Results

A total of 40 patients, 20 (50%) cases and 20 (50%) controls, were enrolled in this study. Clinical symptoms were almost similar in both groups except for dyspnea (80% vs. 40%) and hemoptysis (75% vs. 35%), which were significantly predominant in the case group. Lower zone involvement in chest X-ray was significantly (p = 0.0079) more in the case group (75%) compared to the control group (40%). Cavitary lesions were also significantly higher in the TB with DM group (p = 0.031). Bilateral lesions and multiple zone involvement were also more common in the case group, although no statistically significant difference was seen. Additionally, the hematological parameters of the two groups differed; however, the findings were not statistically significant.

Conclusions

Based on our findings, we recommend screening all TB patients for DM. Similarly, all high-risk DM patients should be screened for TB for early diagnosis and management, thereby reducing morbidity and mortality. Physicians should be aware that people with DM may present with pulmonary TB in an atypical manner.

## Introduction

One of the most common illnesses in the world is tuberculosis (TB), an infectious disease that spreads via the air and is caused by *Mycobacterium tuberculosis*. TB is a global health concern. According to the World Health Organization, approximately 10.6 million individuals globally were diagnosed with TB in 2021, and 1.6 million of those patients lost their lives to the illness [1]. India, with 28% of TB cases, was one of the eight nations in the world that accounted for more than one-third (68.3%) of all TB patients. About three-quarters of all TB-related fatalities worldwide among human immunodeficiency virus (HIV)-negative individuals occurred in India [1]. Diabetes mellitus (DM), one of the oldest known metabolic diseases, affects young as well as elderly people and weakens the immune system [2]. The American Diabetes Association (ADA) describes DM as a collection of metabolic disorders marked by increased blood sugar levels because of deficiencies in the production of insulin, its action, or both [3]. A systemic review of 13 observational studies (n = 1,786,212 participants, and 17,698 TB cases) by Jeon and Murray showed that DM was significantly associated with an increased risk of TB (relative risk = 3.11, 95% confidence interval = 2.27-4.26) compared to non-diabetic people despite differences in study designs and geographic areas [4]. Several other studies done in India alongside a wealth of available literature have also consistently reported evidence for an increased incidence as well as risk of development of TB among people with DM [5-7].

Numerous factors, such as the severity of the sickness and the state of the host’s immune system, affect how TB appears on radiographs. The superior segments of the lower lung lobes and the apical and posterior segments of the upper lung lobes are the most frequently affected by the lesions of post-primary TB, the most prevalent kind of pulmonary TB in adults [8]. In 1927, Sosman and Steidl reported that TB patients with DM had more lower lung involvement while those without DM had more upper lung involvement [9]. Over the years, an abundance of evidence has established a profound influence of DM on both clinical and radiological manifestations in pulmonary TB [2,8,10-13]. However, it is worth noting that conflicting results have also been reported, underscoring the complexity of this interplay. Multiple studies have consistently yielded inconclusive evidence, revealing no significant disparity in lower lung involvement between pulmonary TB patients with DM and their non-diabetic counterparts [14-17]. As a result, it is difficult to draw a firm conclusion owing to the disparate outcomes observed across the reported studies.

With this background, this study was undertaken to investigate, assess, analyze, and compare clinical manifestations, symptoms, and radiological features observed in chest X-rays of patients diagnosed with drug-sensitive pulmonary TB both with and without DM.

## Materials and methods

This comparative, observational study was conducted among pulmonary TB patients in the Department of Respiratory Medicine, Civil Hospital, Ahmedabad from August 2023 to October 2023. All new outpatients and inpatients newly diagnosed with microbiologically confirmed drug-sensitive pulmonary TB, aged above 15 years, who provided written consent to participate in this study voluntarily were included. TB diagnosis was done per the guidelines of the National Tuberculosis Elimination Program (NTEP) [18]. Patients having drug-resistant pulmonary TB, those on immunosuppressant drugs, those with evidence of extrapulmonary TB, individuals having HIV, and those who did not provide informed consent to participate in this study were excluded.

All enrolled patients were split into the following two groups: the control group included drug-sensitive pulmonary TB patients without DM and the case group included patients who either previously had DM or had been diagnosed with DM concurrently with the diagnosis of drug-sensitive pulmonary TB. DM was diagnosed according to the ADA guidelines [3].

All patients who met the inclusion criteria were enrolled in this study after explaining the purpose of this study and obtaining written informed consent. The study received approval from the Institutional Ethics Committee (approval number: EC/Approval/64/2023/18/09/2023). The data of all included patients were collected using a preformed, structured questionnaire via the interview method. The assessment included demographic details; clinical symptoms, such as cough, fever, chills, night sweats, chest pain, fatigue, hemoptysis, dyspnea, and weight loss; detailed past and family histories of any comorbid conditions, including DM and TB; laboratory investigations; sputum acid-fast bacilli (AFB) results; and chest X-ray findings. Following an extensive physical examination and a thorough history, all patients suspected of having pulmonary TB were subjected to hematological investigations, including complete hemogram, HIV status, fasting blood glucose, and glycosylated hemoglobin (HbA1c), as well as sputum AFB examination and chest posteroanterior view X-ray. Sputum culture and drug susceptibility tests were also performed to rule out drug-resistant TB.

Based on the findings, sputum-positive as well as sputum-negative cases with clinical, radiographic, and culture-positive drug-sensitive pulmonary TB were included in the study. In situations where sputum was negative, radiological findings and culture conversion were used to determine the outcome. Sputum smear examination was graded as scanty, +, ++, or +++, as per the NTEP guidelines [18]. One of the pulmonologists who participated in this study examined each chest X-ray to determine the extent of the disease, the involvement of the zones, and the presence or absence of cavities.

Patients taking insulin or oral hypoglycemic drugs at the time of admission in hospital or were found to have a fasting blood glucose of greater than or equal to 126 mg/dL or HbA1c value of 6.5% or higher were considered to have a diagnosis of DM, as per diagnostic criteria defined by ADA [3].

## Results

This prospective, comparative, observational study included 40 patients who satisfied the exclusion and inclusion criteria. This study was done in equally distributed (1:1), randomized patient groups. In total, 20 (50%) of the 40 patients recruited in the study were cases, while the remaining 20 (50%) were controls. The mean age (±standard deviation) of the entire study population was 42.58 (±16.14) years. The mean age of the case group (49.2 ± 14.68 years) was significantly higher than the mean age of the control group (35.95 ± 15.06 years) (p = 0.0076). There were 10 (50%) male patients and 10 (50%) female patients in the case group, whereas in the control group, male (13, 65%) patients outnumbered female (7, 35%) patients. The total male population in this study was 23 (57.5%), which was slightly higher than the female population (17, 42.5%).

**Figure 1:**
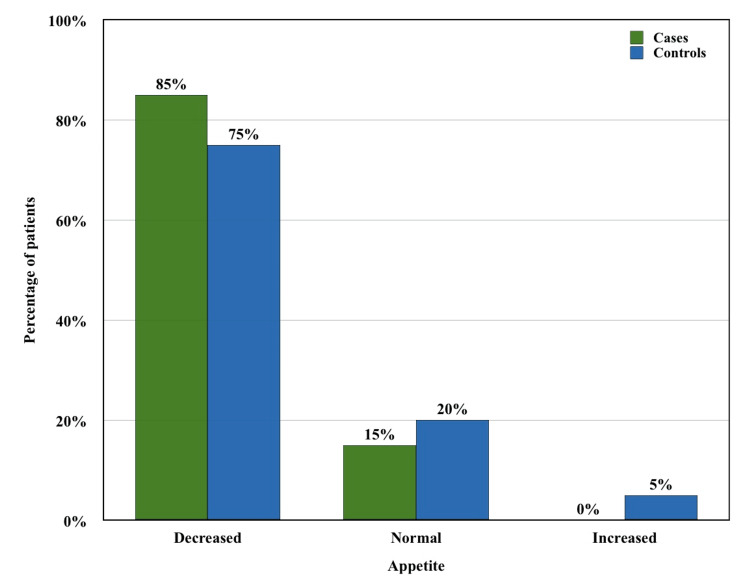
Comparison of presentation of appetite between cases and controls.

In this study, it was observed that clinical symptoms were almost similar in cases and controls except for dyspnea (80% vs. 40%, Fisher’s exact test p-value = 0.0225) and hemoptysis (75% vs. 35%, Fisher’s exact test p-value = 0.0248), which were significantly predominant in the case group (Table 1). In addition, in the case group, 17 (85%) patients had decreased appetite compared to 15 (75%) in the control group (Figure 1). However, there was no statistically significant difference (Fisher’s exact test p-value = 0.6948). In the case group, eight (40%) patients reported a decrease in sleep, and six (30%) patients reported an increase in sleep, while in the control group, seven (35%) and four (20%) patients had decreased and increased sleep, respectively. Six (30%) patients in the case group and four (20%) patients in the control group had a history of smoking.

**Table 1:** Clinical symptoms presentation in cases and controls.

Clinical symptoms	Case group (20)	Control group (20)	Total (40)	P-value (Fisher’s exact test)
Cough	16 (80%)	18 (90%)	34 (85%)	0.6614
Fever	19 (95%)	17 (85%)	36 (90%)	0.605
Dyspnea	16 (80%)	8 (40%)	24 (60%)	0.0225
Chills	10 (50%)	10 (50%)	20 (50%)	1.0000
Night sweats	12 (60%)	7 (35%)	19 (47.5%)	0.2049
Hemoptysis	15 (75%)	7 (35%)	22 (55%)	0.0248
Fatigue	17 (85%)	17 (85%)	34 (85%)	1.0000
Weight loss	18 (90%)	15 (75%)	33 (82.5%)	0.4075
Chest pain	13 (65%)	9 (45%)	22 (55%)	0.3406

It was observed that two (10%) patients in the case group and three (15%) patients in the control group were negative for sputum AFB at the time of diagnosis. We also observed that 18 (90%) cases had +, ++, or +++ sputum AFB results compared to controls, whereas 14 (70%) patients had +, ++, or +++ sputum AFB findings. However, none of these results were statistically significant (Table 2).

**Table 2:** Difference in sputum AFB status at the time of diagnosis between cases vs. controls. AFB = acid-fast bacilli

Sputum AFB smear	Case group (20)	Control group (20)	Total (40)	P-value (Fisher’s exact test)
Negative	2 (10%)	3 (15%)	5 (12.5%)	1.0000
Scant	0 (0%)	3 (15%)	3 (7.5%)	0.2308
+	6 (30%)	4 (20%)	10 (25%)	0.7164
++	7 (35%)	6 (30%)	13 (32.5%)	1.0000
+++	5 (25%)	4 (20%)	9 (22.5%)	1.0000
+/++/+++	18 (90%)	14 (70%)	32 (80%)	0.2351

In this study, we compared hematological parameters between the two groups (Table 3). However, we could not find any significant difference between any of the hematological results between the two groups except for fasting blood glucose level and HbA1c value which were extremely statistically significantly higher in the case group.

**Table 3:** Difference in hematological investigations in cases vs. controls. SD = standard deviation; TLC = total leukocyte count; ANC = absolute neutrophil count; ALC = absolute lymphocyte count; RBC = red blood cell; Hb = hemoglobin; HbA1c = glycosylated hemoglobin

Hematological tests	Case group (mean ± SD)	Control group (mean ± SD)	t-score	P-value
TLC (×10^3^ cells/mm^3^)	11.0245 ± 2.7593	11.6730 ± 3.8994	0.6071	0.5474
ANC (×10^3^ cells/mm^3^)	8.1141 ± 2.1312	8.7226 ± 3.1962	0.7084	0.4830
ALC (×10^3^ cells/mm^3^)	1.8507 ± 0.6410	1.8440 ± 0.4971	0.0369	0.9707
Platelet count (×10^3^ cells/mm^3^)	288.150 ± 116.643	290.050 ± 77.454	0.0607	0.9519
RBC count (×10^6^ cells/mm^3^)	4.6305 ± 0.6802	4.2590 ± 0.5656	1.8781	0.0681
Hb (g/dL)	12.16 ± 1.98)	11.21 ± 1.40	1.7520	0.0878
HbA1c (%)	9.2250 ± 2.0447	4.5915 ± 1.0164	9.0749	<0.0001
Fasting blood glucose (mg/dL)	221.8185 ± 80.3068	97.3670 ± 13.3467	6.8367	<0.0001

**Figure 2:**
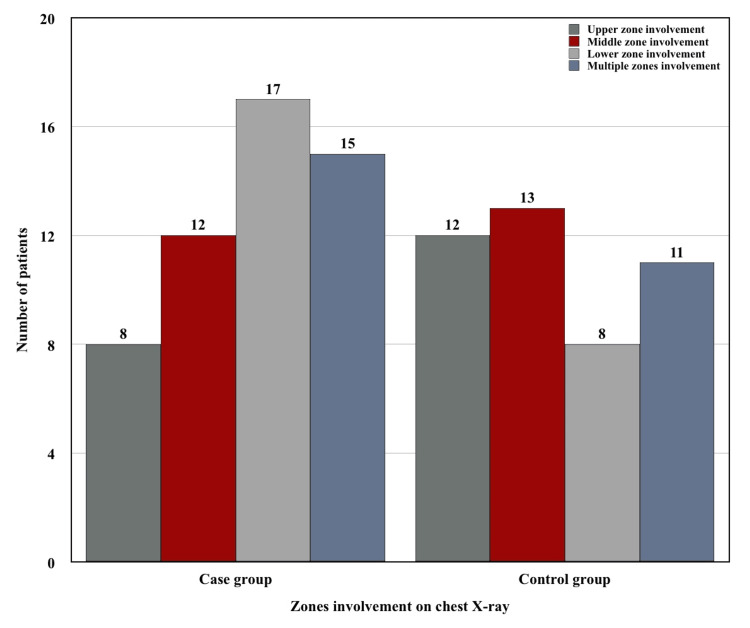
Difference in the involvement of zones on chest X-ray in cases vs. controls.

**Figure 3:**
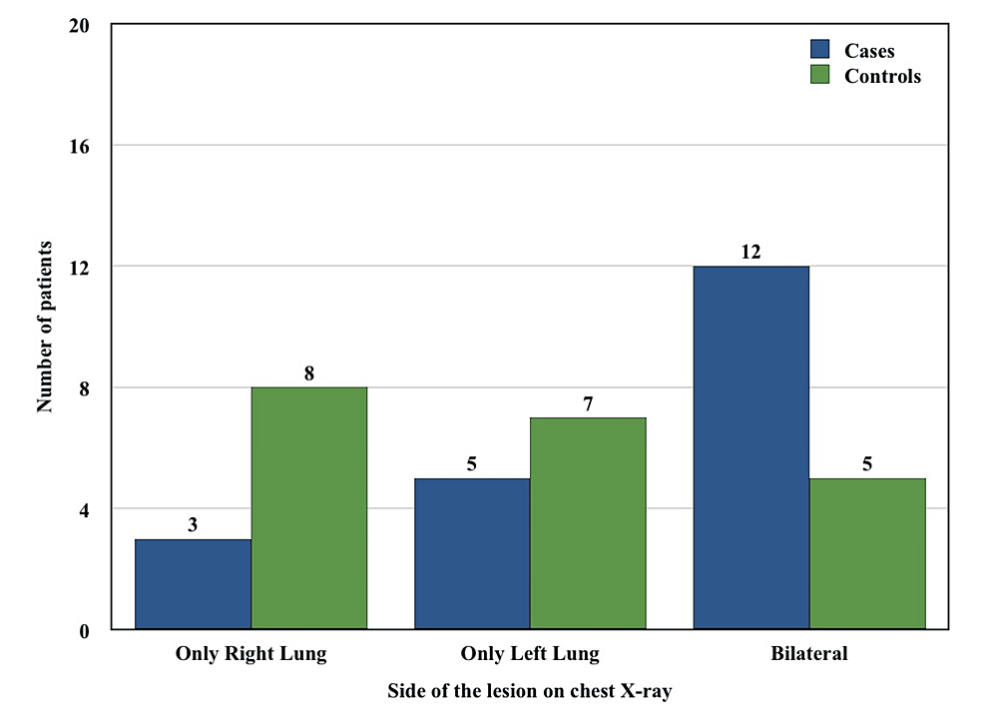
Localization of lesions in chest X-ray in cases vs. controls.

The radiological findings observed in chest X-rays were compared between two groups and the findings are shown in Figure 2 and Figure 3.

It was observed that lower zone involvement in the case group (17/20, 85%) was significantly higher than the control group (8/20, 40%) (Table 4) (Fisher’s exact test p-value = 0.0079). Multiple zone involvement was also more predominant in the case group (15/20, 75%) than in the control group (11/20, 55%) (Figure 2); however, it was not statistically significant (Fisher’s exact test p-value = 0.3203). Furthermore, bilateral lesions were more common in the case group (12/20, 60%) compared to the control group (5/20, 25%) (Figure 3), although this result was not statistically significant (Fisher’s exact test p-value = 0.0536). We also noticed more cavitary lesions in the case group (9/20, 45%) compared to the control group (2/20, 10%), which was statistically significant (Table 5) (Fisher’s exact test p-value = 0.031).

**Table 4:** Lower zone involvement in chest X-ray among cases vs. controls. Fisher’s exact test p-value = 0.0079; statistically significant at p < 0.05.

Involvement of the lower zone in chest X-ray	Case group (20)	Control group (20)	Total (40)
Present	17 (85%)	8 (40%)	25 (62.5%)
Absent	3 (15%)	12 (60%)	15 (37.5%)
Total	20 (100%)	20 (100%)	40 (100%)

**Table 5:** Significance of chest X-ray cavitary lesions presentation between cases vs. controls. Fisher’s exact test p-value = 0.031; statistically significant at p < 0.05.

Cavitary lesion	Case group (20)	Control group (20)	Total (40)
Present	9 (45%)	2 (10%)	11 (27.5%)
Absent	11 (55%)	18 (90%)	29 (72.5%)
Total	20 (100%)	20 (100%)	40 (100%)

## Discussion

Numerous studies conducted over the years have demonstrated the significant impact DM has on the clinical and radiological manifestations of pulmonary TB. In this study, 20 drug-sensitive pulmonary TB patients with DM (case group) were compared with 20 drug-sensitive pulmonary TB patients without DM (control group). We found significantly (p = 0.0076) higher mean age in the TB with DM group (49.2 ± 14.68 years) than in the TB without DM group (35.95 ± 15.06 years). This can be explained by the fact that DM is a disease of middle age and is usually seen in people aged above 40 years. Similar results were seen in other previously published studies [11,19-21]. There were 23 (57.5%) male patients and 17 (42.5%) female patients in this study. The male preponderance can be explained by the fact that most developing countries, including India, have higher rates of social and occupational exposure for males, which promotes the spread of TB. Another factor might be the greater incidence of underdiagnosis in women, which is mostly because of fewer opportunities to obtain medical services in women. Similar results were seen in other studies [2,10,19,21].

We found similar symptoms in the TB with DM group and the TB without DM group except for dyspnea and hemoptysis, which were significantly greater in the TB with DM group. Our findings were similar to various studies done in the past [10,11,20]. However, some studies showed opposite results [2,16]. One study showed a similar result for hemoptysis as ours but reported an opposite result regarding dyspnea [17]. We also observed that 17 (85%) patients in the TB with DM group had appetite loss compared to 15 (75%) patients in the TB without DM group. However, this finding was statistically insignificant in our study. We found that two (10%) patients in the TB with DM group and three (15%) patients in the TB without DM group were negative for sputum AFB at the time of diagnosis. Although this result was not statistically significant in our study, it was similar to the results observed in other studies which reported a significantly higher frequency of negative sputum AFB in the TB without DM group [2,10].

In our study, we compared hematological parameters between the two groups (Table 3). Our data showed that the mean absolute neutrophil count in the TB with DM group was 8.1141 ± 2.1312 × 10^3^ cells/mm^3^ and 8.7226 ± 3.1962 × 10^3^ cells/mm^3^ in the TB without DM group. The mean absolute lymphocyte count was 1.8507 ± 0.6410 × 10^3^ cells/mm^3^ in the case group and 1.8440 ± 0.4971 × 10^3^ cells/mm^3^ in the control group. We also observed that five (25%) patients in the case group and three (15%) patients in the control group had a platelet count greater than 350 × 10^3^ cells/mm^3^ with a mean platelet count of 288.150 ± 116.643 × 10^3^ cells/mm^3^ in the case group and 290.050 ± 77.454 × 10^3^ cells/mm^3^ in the control group. The mean red blood cell count was 4.6305 ± 0.6802 × 10^6^ cells/mm^3^ in the case group, while in the control group, it was 4.2590 ± 0.5656 × 10^6^ cells/mm^3^. The mean hemoglobin value was 12.16 ± 1.98 g/dL in the case group and 11.21 ± 1.40 g/dL in the control group. Our findings were overall consistent with a study that compared hematological findings between TB patients and healthy controls and reported that platelet count and granulocyte count were significantly higher and lymphocyte count and hemoglobin value were significantly lower in TB patients compared to the normal range [22]. However, we could not find any statistically significant difference between the TB with DM group and the TB without DM group in any of the above hematological parameters.

Our study reported that lower zone involvement in chest X-ray was significantly higher (Fisher’s exact test p-value = 0.0079) in pulmonary TB with DM (17/20, 85%) compared to pulmonary TB without DM group (8/20, 40%). Multiple previous studies showed the same atypical finding in chest X-rays [2,8-13,23,24]. However, several other studies showed similar radiological findings in both groups [14-17,25]. Our study also showed significantly more cavitary lesions in cases compared to controls. This finding was quite similar to the results of various other studies [8,10,13,17,20].

## Conclusions

In this study, we found that DM influences the clinical manifestations and radiological characteristics of pulmonary TB. Hemoptysis and dyspnea, cavitary lesions, and lower zone involvement in chest X-rays were significantly predominant in TB with DM patients. Our study also reported differences in hematological parameters between the two groups. Based on our findings, we recommend screening all TB patients for DM, and, similarly, all high-risk DM patients should be screened for TB for early diagnosis and management, thereby reducing morbidity and mortality. Physicians should be aware that DM patients may present with pulmonary TB in an atypical manner.
